# Microbiological Profiles of Patients with Spondylodiscitis

**DOI:** 10.3390/antibiotics13070671

**Published:** 2024-07-19

**Authors:** Frank Sebastian Fröschen, Pia Maria Kitkowski, Andreas Christian Strauß, Ernst Molitor, Gunnar Thorben Rembert Hischebeth, Alexander Franz

**Affiliations:** 1 Department of Orthopaedics and Trauma Surgery, University Hospital Bonn, 53127 Bonn, Germany; 2 Institute of Medical Microbiology, Immunology and Parasitology, University Hospital Bonn, 53127 Bonn, Germany; molitor@uni-bonn.de (E.M.); hischebeth@microbiology-bonn.de (G.T.R.H.)

**Keywords:** spine, spondylodiscitis, infection, antimicrobial resistance, microorganism, antibiotic stewardship, antimicrobial treatment

## Abstract

Spondylodiscitis is a severe spinal infection that requires an effective antibiotic treatment. Therefore, we sought to analyse the causative pathogens from intraoperative specimen in patients with spondylodiscitis and a need for surgery. To this end, we performed a retrospective study of all patients with spondylodiscitis and a need for operative treatment admitted to our hospital between January 2020 and December 2022. A total of 114 cases with spondylodiscitis were identified. A total of 120 different pathogens were detected. Overall, 76.7% of those microorganisms were Gram-positive bacteria. The most common causative pathogen was *Staphylococcus aureus* (*n* = 32; 26.6%), followed by coagulase-negative staphylococci (*n* = 28; 23.4%), of which *Staphylococcus epidermidis* (*n* = 18; 15%) was the most frequently detected, as well as enterococci (*n* = 10; 8.4%) and *Streptococcus* spp. (*n* = 11; 9.2%). Moreover, 19.1% (*n* = 22) and 3.4% (*n* = 4) of all detected isolates were Gram-negative pathogens or fungi, respectively. Overall, 42.8% of all coagulase-negative staphylococci were oxacillin-resistant, while none of them were vancomycin-resistant. In summary, 50% of the pathogens could be identified as staphylococci. The results of our study highlight the important burden of oxacillin-resistant Gram-positive bacteria as an aetiological cause of spondylodiscitis, providing a relevant finding for antimicrobial stewardship programmes.

## 1. Introduction

Diagnosing and treating spondylodiscitis remains one of the most challenging situations for health care professionals in Europe. Conventional radiological imaging of the relevant spinal segments is often used as a first-line diagnostic option in patients with persistent or severe spinal symptoms and increased inflammatory markers [1,2]. Although the sensitivity (82%) and specificity (57%) of conventional radiological imaging are low, its broad availability is the main advantage [3]. A spinal infection must be considered in the presence of typical radiographic features such as erosion of the base and/or end plate, as well as destruction of the vertebral body. The gold standard for diagnosing or ruling out a spinal site infection remains magnetic resonance imaging (MRI)—if possible, gadolinium-enhanced—with 92% sensitivity and 96% specificity [1,4]. In addition, MRI enables visualisation of the extent of the infection and possible abscess formations [5]. Contrast-enhanced computed tomography (CT) is a possible alternative in patients with contraindications to MRI (e.g., old non-MRI-compatible pacemakers). Nevertheless, mean time to diagnosis of spondylodiscitis from the onset of symptoms is reported to vary from 35 to 50 days [6,7].

After diagnosing a spinal infection, the main aim of treatment is elimination of the causative pathogen with or without surgery. Therefore, detecting and identifying the causative pathogen is crucial in order to initiate targeted and effective antibiotic therapy [1]. As antibiotic stewardship plays a decisive role in the treatment of infections regardless of the site of infection, empiric therapy should only be continued until the causative pathogen has been identified. Nevertheless, for the best possible care of the patient, initiation of a targeted antibiotic therapy should begin without relevant delay after the diagnosis of a spinal infection [8]. Therefore, knowledge of the local microbiological profiles and data regarding the antimicrobial resistances of various pathogens might be helpful [6,9]. The prevalence of causative pathogens and their antibiotic resistances may vary drastically; for example, *Mycobacterium tuberculosis* is the most common pathogen of spondylodiscitis worldwide, while *Staphylococcus aureus*, followed by Gram-negative pathogens, are the most frequently detected pathogens in Europe [1,2,6].

As the causative pathogen of any given case of spondylodiscitis remains to be identified at the onset of therapy, epidemiological data about the distribution and resistance of pathogens could be helpful in selecting the most suitable antibiotic for empiric therapy. 

The purpose of this study was to characterize the causative pathogens of spinal infections in patients undergoing surgery, evaluate detection rates, and describe the antibiotic susceptibility of the most common pathogens in our institution. 

## 2. Results

A total of 116 patients with spondylodiscitis and an indication for operative treatment who were admitted to our hospital between January 2020 and December 2022 were included in our study. All 114 patients underwent surgery for various reasons. Two patients suffered cardiopulmonary instability during anaesthetic induction and were subsequently deemed unsuitable for surgery and excluded from this study. The clinical characteristics and comorbidities of all patients who underwent surgery are presented in Table 1. The most common comorbidities in patients with spondylodiscitis were hypertension (52.6%), smoking (27.2%) and diabetes mellitus (22.8%). The total number of surgeries performed at our hospital each year was 46 (2020; 41.1%), 36 (2021; 32.1%) and 30 (2022; 26.8%). 

The majority of patients had an acute onset of their symptoms and persistent pain thereafter. Nevertheless, in 21.4% of cases, time until diagnosis of spondylodiscitis was more than 6 weeks. All patients underwent X-ray imaging and MRI for preoperative diagnosis. Blood cultures were taken preoperatively from all patients. Subgroup analysis of patients with a need for emergency surgery and those who chose elective surgery revealed that the patients who chose elective surgery were more likely to be immunosuppressed, suffer with ongoing malignancies or have a neurological deficit existing for more than 24 h.

Pathogens were identified in 15.8% of blood cultures, representing 23 of 114 patients. In all 23 cases, only one pathogen was detected. The most frequently detected pathogen from preoperative blood cultures at admission was *S. aureus* (*n* = 11), followed by *Staphylococcus epidermidis* (*n* = 6), *Escherichia coli* (*n* = 4), *Enterococcus faecalis* (*n* = 1) and *Streptococcus* species (*n* = 1).

In contrast, we identified the causative pathogen in 78.9% (*n* = 90) of cases from the tissue samples that we obtained intraoperatively. Subgroup analysis revealed that in patients with an imminent need for surgical therapy (e.g., during night-time or on weekends; *n* = 62; 54.4%), the detection rate of pathogens from intraoperative specimens was 85.5% (53 of 62 patients), while in patients who chose elective surgery, it was 67.3% (35 of 52 patients).

In summary, 90 patients had intraoperative tissue samples that were positive for microbial pathogens. A total of 120 different pathogens were detected. In 70 of those 90 patients (77.8%), only one pathogen was detected. In 13 of those 90 patients (14.5%), two different pathogens were detected. Three different pathogens were detected in 4 of 90 patients (4.4%), and five different pathogens were detected in 2 of 90 patients (2.2%). The most frequently detected isolates in those 20 patients with polymicrobial infection were *S. epidermidis* (*n* = 8), *Streptococcus* spp. (*n* = 8) and *E. coli* (*n* = 5).

The distribution of all pathogens detected over the analysed three-year period is displayed in Table 2. Overall, 76.7% of all detected microorganisms (*n* = 120) from tissue samples were Gram-positive bacteria, followed by Gram-negative bacteria (19.1%). The most frequently detected pathogens were *S. aureus* (*n* = 32; 26.7%), coagulase-negative staphylococci (CNS; *n* = 28; 23.4%), *E. coli* (*n* = 13; 10.9%) and *Streptococcus* spp. (*n* = 11; 9.2%). Subgroup analysis of the CNS yielded *S. epidermidis* (*n* = 18; 15.0%), *Staphylococcus capitis* (*n* = 5; 4.2%), *Staphylococcus capare* (*n* = 2; 1.7%), *Staphylococcus haemolyticus* (*n* = 2; 1.7%) and anaerobic growing *Staphylococcus saccarolyticus* (*n* = 1; 0.8%).

Table 3 displays the results of the antibiotic susceptibility testing for coagulase-negative staphylococci, *S. aureus*, *Streptococcus* spp. and Gram-negative bacteria. 

In particular, no *S. aureus* displayed resistance to oxacillin, rifampicin or vancomycin. Only one isolate displayed resistance to clindamycin. In contrast, CNS showed an average oxacillin resistance rate of 42.8% (range: 28.6–53.8%). Rifampicin resistance was detected in one isolate (3.5%), while resistance against vancomycin was not detected in any isolated strains. Resistance to clindamycin was detected in CNS in 39.2% of all isolates.

For *Streptococcus* spp., no isolate displayed penicillin resistance. Gram-negative organisms showed an overall resistance rate of 21.7% to both piperacillin–tazobactam and sulfamethoxazole–trimethoprim. Ciprofloxacin resistance of Gram-negative organisms was only detected in one isolate (4.3%), while no isolates displayed resistance to meropenem.

In summary, there were no *S. aureus* isolates or *Streptococcus* spp. with oxacillin or penicillin resistance.

## 3. Discussion

Spondylodiscitis is a severe spinal infection that requires an effective antibiotic treatment and, in severe cases, surgical therapy. Therefore, a fast diagnosis is essential [1]. Reviewing patient history and symptoms is mandatory but not very sensitive or specific for diagnosing a spinal site infection [8]. An evaluation of blood tests, including leukocyte count and CRP, is easy to perform, and in the case of an acute disease, increased inflammatory markers can be found in 75–98% of all patients, according to Cheung et al. [10]. Elevated CRP and leukocytosis often shorten the time to diagnosis in patients with back pain [7].

After diagnosing spondylodiscitis, identification of the causative pathogen is needed. Ideally, before starting empiric antibiotic therapy, blood cultures (at least two pairs: aerobic/anaerobic) should be obtained, which might be helpful in the identification of the causative pathogen or the focus of the infection (e.g., bacterial endocarditis) and should therefore be part of the diagnostic workup [5]. Nevertheless, detection rates of causative pathogens remain low. In the literature, detection rates of pathogens from preoperative blood cultures vary between 30% and 70%, which is higher than our detection rate of 15.8% [1,5]. Interestingly, we were not able to detect polymicrobial infections, a fact debated for the last thirty years, which highlights the importance of identifying pathogens using other methods in addition to blood cultures [11].

Therefore, the most reliable method remains surgical biopsy. According to Fleege et al., the detection rates of pathogens from intraoperative specimens vary between 68% and 93% [12]. In our cohort, we had an overall detection rate of causative pathogens from intraoperative specimens of 78.9% (emergency surgery: 85.5%; elective surgery 67.3%), which are therefore in line with the literature. The differences in intraoperative findings between emergency and elective surgery could be explained by a previously administered intravenous or oral antibiotic therapy. Unfortunately, we were not able to evaluate the exact preoperative treatment in all patients who chose elective surgery.

As spondylodiscitis is very heterogeneous in terms of severity, there is no gold standard for treatment. According to Herren et al., an intervention is recommended in cases of neurological deficits, sepsis, the presence of an intraspinal empyema, the presence of a ventral or paravertebral abscess >2.5 cm, a failure of conservative therapy or spinal instability (segmental kyphosis (>15°), vertebral body collapse (>50%) or translation (>5 mm)) [1]. Here, we must admit that the transfer of patients to our clinic is only performed if at least one of these criteria is fulfilled, which might lead to a selection bias. This is underlined by the very high number of patients with polymicrobial infections (17.5%; 20 of 114 patients) compared to the rates described in the literature, which range from 8% to 15% [6,13]. It must be kept in mind that 75% of all patients with a spinal infection do not undergo surgery, so several polymicrobial infections might still go unnoticed [1,10,12]. In addition, patients sometimes refuse to undergo emergency surgery for personal reasons, despite the presence of a severe spinal infection (e.g., epidural abscess). Hadjipavlo et al. performed biopsies in all patients with a spinal infection—regardless of whether they received surgical or conservative therapy—and detected polymicrobial infections in 24% of all cases, which supports our conclusion [14].

In our study the most frequently detected pathogens were Gram-positive bacteria (76.7%), followed by Gram-negative bacteria (19.1%). This distribution lines up with the findings of Doutchi et al. (2015) in which the detection rate of Gram-positive pathogens was 76% and the detection rate of Gram-negative pathogens was 18% in a French cohort of 50 cases [6]. Herren et al. and Flege et al. stated that, in this context, *S. aureus* was the most relevant pathogen in more than 50% of all cases [1,4,12]. Subgroup analysis of our series showed that *S. aureus* is the most frequently detected pathogen, with a detection rate of 26.6%. Doutchi et al. detected *S. aureus* in 36% of their patients [6]. Therefore, detection rates of *S. aureus* might vary, but *S. aureus* is still the most frequently detected pathogen [15]. In order to treat an *S. aureus* infection, the antibiotic profiles are relevant. In our series, none of the detected isolates displayed methicillin resistance; therefore, cephalosporins (e.g., Cefazolin) or isoxazolylpenicillins (e.g., Flucloxacillin) might be used for targeted antibiotic therapy, whereas in cases of high methicillin resistance rates, vancomycin should be used. Interestingly, MRSA rates of up to 17% have been described [6].

The second-most frequently detected pathogens in patients with spondylodiscitis were CNS (23%), with *S. epidermidis* (15%) being the most common. The role of CNS as causative pathogens in patients with periprosthetic joint infection (PJI) of the hip or knee joint has been well described, and its importance has been outlined elsewhere [9]. In patients with spondylodiscitis, lower detection rates of CNS have been described, which vary between 3% and 16% [2,6,16,17]. Nonetheless, our finding that *S. epidermidis* is the most frequently detected CNS is consistent with the literature [2]. Our oxacillin resistance rate of 42.8% for CNS is consistent with that described in the literature (45%) [6]. To cover those oxacillin-resistant CNS, vancomycin might be administered as empiric therapy.

The detection of Gram-negative bacteria was possible in 22 patients (19.1%), with *E. coli* being the most frequently detected pathogen, followed by *E. cloacae* complex and *K. pneumoniae*. Although *E. coli* is frequently described as the most frequently detected pathogen, with detection rates of up to 11%, there are studies describing *K. pneumoniae* (12.3%) or *P. aeruginosa* (9.2%) as the most frequently detected pathogens [1,6,16,18]. This highlights the importance of targeted antibiotic therapy.

This is confirmed by our results. The resistance of Gram-negative pathogens to common first-line antibiotics was 21.7% for piperacillin–tazobactam, while resistance to ciprofloxacin was only present in one isolate (4.3%). Interestingly, none of the isolates displayed resistance to meropenem. Therefore, in septic patients with spondylodiscitis and suspected Gram-negative pathogens, an initial antibiotic therapy with meropenem should be considered. Our data suggest that an empiric antibiotic treatment with piperacillin–tazobactam has a high risk of treatment failure in the case of Gram-negative spondylodiscitis, provided the causative pathogen is not detected during treatment and antibiotic susceptibility is confirmed or antibiotic treatment—in the case of a resistant pathogen—is not changed.

Another frequently detected group of pathogens is the *Streptococcus* spp. In the literature, detection rates vary between 5% and 20% [2,14]. In their cohort of 31 patients with spondylodiscitis and positive blood cultures, Chelsom et al. described *Streptococcus* spp. as the second most frequently detected pathogens [15]. In contrast, relevant antibiotic resistances are rarely described, a finding consistent with our results, as 9.2% of all isolates were *Streptococcus* spp. [6]. Thankfully, no detected isolates displayed resistance to penicillin, so empiric therapy with Cephalosporins or Aminopenicillins ought to be sufficient.

In contrast to bacterial spondylodiscitis, fungal infections of the spine are still a rare finding [19]. Detection rates of up to 6% have been described [16]. However, these cases are burdened with huge difficulties regarding management and eradication, as patients are often immunocompromised and have relevant comorbidities like diabetes [20]. In our study, only four isolates of Candida spp. (2 × *C. albicans*, 2 × *C. parapsilosis*) were detected in tissue samples from patients with spondylodiscitis, therefore representing a minority. As *Candida* spp. are part of the skin flora, special care should be taken regarding antibiotic and antifungal therapy in these patients. Interestingly, none of the patients in our study with *Candida* spp. in their tissue sample was taking immunosuppressive drugs, a known drug abuser, or had a history of active malignancy. However, all patients with *Candida* spp. suffered from a polymicrobial infection. Our findings are therefore in accordance with the current literature. As there are, at the moment, no radiological findings that can help to differentiate between pathogens, it is important that medical providers be aware of a possible increased risk of fungal infection in patients with polymicrobial infections [19].

Although polymicrobial spinal infections are rare, they remain challenging to treat, and consequently, knowledge of the microbiological spectrum might be useful for treatment. Our most important finding was that *S. epidermidis,* followed by *Streptococcus* spp. and *E. coli,* were the most frequently detected isolates in cases of polymicrobial infection. *S. epidermidis* and *E. coli* were the pathogens most frequently detected in combination (*n* = 2), a finding that is consistent with the literature, although the low number of cases does not allow a recommendation for antibiotic therapy [6,14]. Further studies are needed for a better understanding of pathogen combination in patients with polymicrobial spinal infections.

Our study has some limitations, as we were not able to access the previous microbiological results of external laboratories or the exact history of oral antibiotic therapies that patients might have been treated with before admission to our hospital (e.g., in the case of a respiratory infection). In addition, testing for *Mycobacterium* was only performed in selected cases in which the presence of risk factors for an infection existed. Due to the low prevalence of infections with *Mycobacterium* in Germany, we did not perform cultures for *Mycobacterium* in all patients. As a result, there might be collection and selection biases. Moreover, the data were collected at a level 1 spine surgery center, where patients are often transferred because of complications (e.g., multimorbidity or relevant spinal deformity cause by spinal infection). Here, we would like to point out that discussing the indication for surgery was not the topic of this study. In addition, we did not include patients with conservative therapy. Furthermore, the high number of included patients makes this study one of the largest single-center studies.

## 4. Materials and Methods

In this retrospective study, we included all consecutive cases of patients who underwent surgical treatment for spondylodiscitis between January 2020 and December 2022 at a level 1 spine surgery center in Germany. There were no exclusion criteria.

A diagnosis of spondylodiscitis was based on clinical findings, radiological features and laboratory results. According to our treatment algorithm, X-ray imaging, as well as an MRI, was performed in all patients with a suspected spinal infection. A gadolinium-enhanced MRI was used to confirm diagnosis and distinguish an infection from degenerative changes or neoplasms. In the case of a contraindication for MRI, a computed tomography (CT) scan with contrast agent was performed.

For a better description of the included patients, we recorded patient demographics such as weight and comorbidities. If patients underwent spine surgery several times during this study, only the first surgical intervention was recorded. A polymicrobiological spondylodiscitis was defined as the presence of two or more different microorganisms isolated from the intraoperative tissue biopsies or fluid of one patient. The microbiological profiles of all pathogens were analysed.

Intraoperatively collected tissue specimens were shredded, homogenized and then plated on Columbia agar with 5% sheep blood, MacConkey agar, chocolate agar and Sabouraud agar (Becton, Dickinson and Company, Bergen County, NJ, USA), and 1 mL was pipetted into thioglycolate bouillon (Becton, Dickinson and Company, Bergen County, NJ, USA).

For anaerobic cultures, Schaedler and kanamycin/vancomycin agar plates (Becton, Dickinson and Company, Bergen County, NJ, USA) were struck with shredded and homogenized tissue specimens. All cultures were grown at 5% CO_2_ and 35 °C for at least 14 days. Fluid aspirates were inoculated in PEDS medium blood culture flasks (Becton, Dickinson and Company, Bergen County, NJ, USA) and incubated in a Bactec FX blood culture system (Becton, Dickinson and Company, Bergen County, NJ, USA) for 14 days. If requested, a culture for *Mycobacterium* was also performed. Only patients with the following risk factors were tested for *Mycobacterium*: a migrant background from high-prevalence countries according to the WHO global tuberculosis reports, a long-term visit to countries with a high prevalence of tuberculosis, immunosuppression, previous infection with *Mycobacterium* and contact with patients with a known infection of *Mycobacterium*. After appropriate enrichment procedures, liquid and solid cultures were used. A culture period of 12 weeks was also used.

The identification of pathogens was carried out using matrix-assisted LASER desorption-ionization time of flight mass spectroscopy (MALDI-TOF MS; bioMérieux, Nürtingen, Germany). Additionally, antimicrobial susceptibility testing was performed with Vitek2 (bioMérieux, Nürtingen, Germany), an automated antimicrobial susceptibility testing system. In cases in which anaerobic pathogens were detected, susceptibility testing was carried out with a semiautomated microtiter broth dilution system (MICRONAUT; Merlin, Bornheim, Germany). For interpretations of antimicrobial susceptibility, the EUCAST clinical breakpoints (v. 12.0, 2022) were applied.

### Statistical Analysis

Data were collected in Microsoft Excel 2024 (Microsoft Corporation, Richmond, VA, USA). Statistical analysis was carried out with SPSS statistics 28 for Windows (SPSS, Inc., an IBM Company, Chicago, IL, USA). Descriptive statistics, including the arithmetic mean value and the standard deviation, were calculated. Data are given as means, standard deviations (SDs) and ranges, if not indicated otherwise.

## 5. Conclusions

Knowledge of the antibiotic susceptibility of causative pathogens in patients with spondylodiscitis plays an important role in choosing the more appropriate empiric antibiotic therapy. For the detection of causative pathogens, surgical biopsy might be regarded as the gold standard. As the majority of spinal infections are caused by Gram-positive pathogens, with *S. aureus* as the most frequently identified bacterium followed by coagulase-negative staphylococci, oxacillin resistance must be considered. In the case of a septic patient in need of an empiric therapy or who is infected with an unknown pathogen, vancomycin might be the antibiotic of choice. If an infection with Gram-negative bacteria is suspected, additional treatment with piperacillin–tazobactam or meropenem should be considered. Identification of the causative agent (or agents) should always lead to optimized antimicrobial treatment.

## Figures and Tables

**Table 1:** Demographic data and comorbidities of all included patients. * = according to intraoperative finding.

Clinical Features	2020–2022	Emergency Surgery	Elective Surgery
Number of patients	114 (77 Male, 37 Female)	62 (39 Male, 23 Female)	52 (39 Male, 13 Female)
Age [years], mean ± SD (range)	67.3 ± 14.2 (23.4–96.9)	65.3 ± 15.5	69.5 ± 12.4
BMI [kg/m^2^]	29.2 ± 7.9 (18.4–63.8)	28.7 ± 7.8	29.6 ± 8.2
Comorbidities			
Diabetes mellitus	22.8% (*n* = 26)	22.6% (*n* = 14)	23.1% (*n* = 12)
Hypertension	52.6% (*n* = 60)	50% (*n* = 31)	55.8 (*n* = 29)
Smoking	27.2% (*n* = 31)	27.3% (*n* = 17)	26.8% (*n* = 14)
Drugs	5.3% (*n* = 6)	6.5% (*n* = 4)	3.7% (*n* = 2)
Alcoholism	16.7% (*n* = 19)	16.1% (*n* = 10)	17.3% (*n* = 10)
Endocarditis Myocarditis	2.6% (*n* = 3) 0.9% (*n* = 1)	1.6% (*n* = 1) 1.6% (*n* = 1)	3.7% (*n* = 2) 0% (*n* = 0)
Rheumatoid arthritis	5.3% (*n* = 6)	6.5% (*n* = 4)	3.7% (*n* = 2)
Immunosuppression	5.3% (*n* = 6)	1.6% (*n* = 1)	9.6% (*n* = 5)
Malignancy Ongoing Aftercare	12.3% (*n* = 14) 5.3% (*n* = 6) 7.0% (*n* = 8)	8.1% (*n* = 5) 1.6% (*n* = 1) 6.5% (*n* = 4)	17.3% (*n* = 9) 9.6% (*n* = 5) 7.7% (*n* = 4)
Chronic kidney disease	21.9% (*n* = 25)	20.9% (*n* = 13)	23.1% (*n* = 12)
Symptom duration <6 weeks >6 weeks	78.1% (*n* = 89) 21.1% (*n* = 24)	82.3% (*n* = 51) 17.8% (*n* = 11)	73.1% (*n* = 38) 26.9% (*n* = 14)
Preoperative C-reactive protein [mg/L]	188.5 ± 95.4	137.7 ± 98.4	95.1 ± 87.3
Leukocyte count preoperative [G/L]	10.3 ± 5.4	12.1 ± 6.0	8.4 ± 3.7
Preoperative neurological deficit Clinical evaluation not possible	21.1% (*n* = 24) 2.6% (*n* = 3)	14.5% (*n* = 9) 0%	28.9% (*n* = 15) 5.8% (*n* = 3)
Epidural abscess * Prevertebral abscess (e.g., abscess in M. psoas) Epidural abscess and prevertebral abscess	43.8% (*n* = 49) 47.3% (*n* = 53) 23.7% (*n* = 27)	40.2% (*n* = 25) 50.0% (*n* = 31) 24.2% (*n* = 15)	46.2% (*n* = 24) 42.3% (*n* = 22) 23.1% (*n* = 12)
Second vertebral infection site/spondylodiscitis	7.1% (*n* = 8)	8.1% (*n* = 5)	5.8% (*n* = 3)

**Table 2:** Culture data from intraoperative specimens over a three-year period, sorted by organism. *: for a better overview, the Gram classification was used for bacteria, except *Mycobacterium tuberculosis* and *fungi*.

	2020	2021	2022	Total by Species
**Gram-positive bacteria**	43	25	24	83 (76.7%)
*S. aureus*	15	13	4	32 (26.7%)
Coagulase-negative staphylococci	12	7	9	28 (23.4%)
*Streptococcus* spp.	2	2	7	11 (9.2%)
*E. faecalis*	8	1	1	10 (8.4%)
*C. acnes*	5	1	1	7 (5.8%)
*Micrococcus luteus*	0	1	0	1 (0.8%)
*Corynebacterium amycolatum*	1	0	0	1 (0.8%)
*Schaalia odontolytica*	0	0	1	1 (0.8%)
*Parvimonas micra*	0	0	1	1 (0.8%)
**Gram-negative** **bacteria**	5	6	11	22 (19.1%)
*E. coli*	3	3	7	13 (10.9%)
*E. cloacae* complex	2	1	0	3 (2.5%)
*K. pneumoniae*	0	0	3	3 (2.5%)
*P. mirabilis*	0	1	0	1 (0.8%)
*Serratia marcescens*	0	1	0	1 (0.8%)
*P. aeruginosa*	0	0	1	1 (0.8%)
*Prevotella intermedia*	0	0	1	1 (0.8%)
Mycobacterium tuberculosis *	0	1	0	1 (0.8%)
**Yeasts** (*Candida* spp.)	2	0	2	4 (3.4%)
Total by year	50	32	38	120 (100%)

**Table 3:** Antibiotic resistance of selected pathogens against selected antibiotics.

Pathogen		2020		2021		2022		2020–2022		Total
		r	s	r	s	r	s	r	s	
*S. aureus*	oxacillin	0	15	0	13	0	4	0	32	32
	rifampicin	0	15	0	13	0	4	0	32	32
	vancomycin	0	15	0	13	0	4	0	32	32
	clindamycin	1	14	0	13	0	4	1	31	32
Coagulase-negative staphylococci	oxacillin	7	6	2	5	3	5	12	16	28
	rifampicin	0	13	1	6	0	8	1	27	28
	vancomycin	0	13	0	7	0	8	0	28	28
	clindamycin	8	5	2	5	1	7	11	17	28
*Streptococcus* spp.	penicillin G	0	2	0	2	0	7	0	11	11
	clindamycin	2	0	1	1	1	6	4	7	11
Gram-negative bacteria	piperacillin–tazobactam	0	5	0	6	5	7	5	18	23
	ciprofloxacin	0	5	0	6	1	11	1	22	23
	meropenem	0	5	0	6	0	12	0	23	23
	sulfamethoxazole–trimethoprim	2	3	3	3	0	12	5	18	23

r = resistant; s = susceptible; susceptibility testing according to EUCAST.

## Data Availability

The data presented in this study are available in the manuscript.

## References

[1] Herren C., Jung N., Pishnamaz M., Breuninger M., Siewe J., Sobottke R. (2017). Spondylodiscitis: Diagnosis and Treatment Options. Dtsch. Ärztebl. Int..

[2] Gouliouris T., Aliyu S.H., Brown N.M. (2010). Spondylodiscitis: Update on Diagnosis and Management. J. Antimicrob. Chemother..

[3] Grados F., Lescure F.X., Senneville E., Flipo R.M., Schmit J.L., Fardellone P. (2007). Suggestions for Managing Pyogenic (Non-Tuberculous) Discitis in Adults. Jt. Bone Spine.

[4] Herren C., von der Hoeh N.H., Zwingenberger S., Sauer D., Jung N., Pieroh P., Drange S., Pumberger M., Scheyerer M.J. (2023). And Spine Section of the German Society for Orthopaedics and Trauma Spondylodiscitis in Geriatric Patients: What Are the Issues?. Glob. Spine J..

[5] Cornett C.A., Vincent S.A., Crow J., Hewlett A. (2016). Bacterial Spine Infections in Adults: Evaluation and Management. J. Am. Acad. Orthop. Surg..

[6] Doutchi M., Seng P., Menard A., Meddeb L., Adetchessi T., Fuentes S., Dufour H., Stein A. (2015). Changing Trends in the Epidemiology of Vertebral Osteomyelitis in Marseille, France. New Microbes New Infect..

[7] Jean M., Irisson J.-O., Gras G., Bouchand F., Simo D., Duran C., Perronne C., Mulleman D., Bernard L., Dinh A. (2017). Diagnostic Delay of Pyogenic Vertebral Osteomyelitis and Its Associated Factors. Scand. J. Rheumatol..

[8] Sobottke R., Seifert H., Fätkenheuer G., Schmidt M., Goßmann A., Eysel P. (2008). Current Diagnosis and Treatment of Spondylodiscitis. Dtsch. Ärztebl. Int..

[9] Fröschen F.S., Randau T.M., Franz A., Molitor E., Hischebeth G.T.R. (2022). Microbiological Profiles of Patients with Periprosthetic Joint Infection of the Hip or Knee. Diagnostics.

[10] Cheung W.Y., Luk K.D.K. (2012). Pyogenic Spondylitis. Int. Orthop..

[11] Patzakis M.J., Rao S., Wilkins J., Moore T.M., Harvey P.J. (1991). Analysis of 61 Cases of Vertebral Osteomyelitis. Clin. Orthop..

[12] Fleege C., Wichelhaus T.A., Rauschmann M. (2012). Systemische und lokale Antibiotikatherapie bei konservativ und operativ behandelten Spondylodiszitiden. Der Orthopäde.

[13] Vettivel J., Bortz C., Passias P.G., Baker J.F. (2019). Pyogenic Vertebral Column Osteomyelitis in Adults: Analysis of Risk Factors for 30-Day and 1-Year Mortality in a Single Center Cohort Study. Asian Spine J..

[14] Hadjipavlou A.G., Mader J.T., Necessary J.T., Muffoletto A.J. (2000). Hematogenous Pyogenic Spinal Infections and Their Surgical Management. Spine.

[15] Chelsom J., Solberg C.O. (1998). Solberg Vertebral Osteomyelitis at a Norwegian University Hospital 1987-97: Clinical Features, Laboratory Findings and Outcome. Scand. J. Infect. Dis..

[16] D’Agostino C., Scorzolini L., Massetti A.P., Carnevalini M., d’Ettorre G., Venditti M., Vullo V., Orsi G.B. (2010). A Seven-Year Prospective Study on Spondylodiscitis: Epidemiological and Microbiological Features. Infection.

[17] Jensen A.G., Espersen F., Skinhøj P., Frimodt-Møller N. (1998). Bacteremic Staphylococcus Aureus Spondylitis. Arch. Intern. Med..

[18] Kehrer M., Pedersen C., Jensen T.G., Lassen A.T. (2014). Increasing Incidence of Pyogenic Spondylodiscitis: A 14-Year Population-Based Study. J. Infect..

[19] Kim C.W., Perry A., Currier B., Yaszemski M., Garfin S.R. (2006). Fungal Infections of the Spine. Clin. Orthop..

[20] Caldera G., Cahueque Lemus M.A. (2016). Fungal Spondylodiscitis: Review. J. Spine.

